# Bone status in patients with chronic hypoparathyroidism: results from the Italian HypoparaNET database

**DOI:** 10.1007/s40520-025-03139-9

**Published:** 2025-07-23

**Authors:** Francesca Marini, Francesca Giusti, Blandine Weiss, Michel Ovize, Salvatore Benvenga, Filomena Cetani, Annamaria Colao, Sabrina Corbetta, Marta Bondanelli, Maurizio Iacobone, Andrea Lenzi, Giovanna Mantovani, Rosaria Maddalena Ruggeri, Luigi di Filippo, Andrea Giustina, Dalal S. Ali, Aliya A. Khan, Gemma Marcucci, Maria Luisa Brandi, on the behalf of the HypoparaNet team

**Affiliations:** 1 FirmoLab, Fondazione FIRMO Onlus and Stabilimento Chimico Farmaceutico Militare, Florence, Italy; 2 Department of Experimental and Clinical Biomedical Sciences, University of Florence, Florence, Italy; 3 Donatello Bone Clinic, Villa Donatello Hospital, Sesto Fiorentino, Italy; 4 Amolyt Pharma, Ecully, France; 5 Department of Clinical and Experimental Medicine, University of Messina, Messina, Italy; 6 Department of Clinical and Experimental Medicine, Endocrinology Unit, University of Pisa, Pisa, Italy; 7 Department of Clinical Medicine and Surgery, Federico II Naples University, Naples, Italy; 8 Metabolic Bone Diseases and Diabetes Unit, IRCCS Istituto Auxologico Italiano, Milan, Italy; 9 Department of Biomedical, Surgical and Dental Sciences, University of Milan, Milan, Italy; 10 Section of Endocrinology, Geriatrics and Internal Medicine, Department of Medical Sciences, University of Ferrara, Ferrara, Italy; 11 Endocrine Surgery Unit, Department of Surgery, Oncology and Gastroenterology, University of Padua, Padua, Italy; 12 Department of Experimental Medicine, Section Medical Pathophysiology, Endocrinology and Nutrition, University “Sapienza” of Rome, Rome, Italy; 13 Endocrinology Unit, Fondazione IRCCS Cà Granda Ospedale Maggiore Policlinico, Milan, Italy; 14 Department of Clinical Sciences and Community Health, University of Milan, Milan, Italy; 15 Endocrinology Unit, Department of Human Pathology of Adulthood and Childhood DETEV, University of Messina, Messina, Italy; 16 Institute of Endocrine and Metabolic Sciences, San Raffaele Vita-Salute University and IRCCS San Raffaele Hospital, Milan, Italy; 17 Divisions of Endocrinology and Metabolism, McMaster University, Hamilton, ON Canada

**Keywords:** Chronic hypoparathyroidism (HypoPT), Parathyroid hormone (PTH), Bone mineral density (BMD), Bone mass, Osteoporosis, Osteopenia

## Abstract

**Background:**

Chronic hypoparathyroidism (HypoPT) is a rare endocrine condition, having variable etiology, characterized by low parathyroid hormone levels, leading to reduced calcium levels and increased phosphorus values in the blood. Bone health is an important clinical aspect to be considered in patients with HypoPT, whose skeleton is exposed both to the HypoPT-induced alteration of bone mass and microarchitecture, and to the natural occurrence and progression of bone mass loss due to menopause and ageing.

**Aim:**

Investigating bone status in a cohort of Italian HypoPT patients from the HypoparaNET database.

**Methods:**

In this cross-sectional study, we retrospectively analyzed bone status in 162 adult, and 11 children and adolescent HypoPT cases, based to their sex, age, HypoPT etiology, urinary excretion of calcium and phosphate, and serum level of 25(OH)-vitamin D.

**Results:**

Overall prevalence of osteoporosis in adult HypoPT patients was found to be slightly lower to that of the general population (17.3% vs 18.3%), increasing with ageing and being more frequent in postmenopausal women. Data from our HypoPT cohort confirmed that ageing and female sex are independent risk factors for osteoporosis, even among individuals with HypoPT.

**Discussion:**

Results from this study suggest the importance of a regular follow-up of bone health in HypoPT, especially in postmenopausal women and young patients with a genetic form of HypoPT.

**Conclusions:**

Medical therapies aim at restoring a balanced bone turnover and preventing bone tissue loss could be indicated in HypoPT cases with reduced bone mass to grant a better bone health at any age.

## Introduction

Chronic hypoparathyroidism (HypoPT) is a rare endocrine disorder characterized by hypocalcemia and hyperphosphatemia, due to persistent absence or severe deficiency of parathyroid hormone (PTH). HypoPT is prevalently (approximately 75% of cases) an acquired disease, caused by neck surgery or irradiation, removing or disrupting parathyroid glands. Post-surgical HypoPT is defined as a permanent hypocalcemic condition lasting more than 12 months after neck surgery [1]. The remnant 25% of HypoPT cases overall includes autoimmune destruction or infiltration of parathyroid glands, genetically-driven congenital defects of parathyroid development or activity, HypoPT secondary to chronic hypomagnesemia, and idiopathic forms [2].

Information regarding bone health in HypoPT patients of all ages are currently limited, and this clinical aspect is not routinely investigated in the management of these patients. PTH is a key regulator of bone remodeling rate, and the chronic deficiency/absence of this hormone in HypoPT patients causes profound reduction in bone remodeling [3], leading to increased bone mineral density (BMD), changes in bone microarchitecture, and alteration in bone strength. Typically, BMD, evaluated by Dual-energy X-ray Absorptiometry (DXA), is greater in both male and female HypoPT patients, than in normal subjects of the same age and sex, presumably due to low bone turnover [3–5]. HypoPT was also shown to slow the expected rate of bone loss in postmenopausal women [6]. Bone evaluation by peripheral quantitative computed tomography (pQCT) showed greater trabecular and cortical volumetric BMD (vBMD), higher trabecular number, and lower total bone area at both periosteal and endosteal surfaces in HypoPT patients, than both individuals with normal parathyroid function and patients with primary hyperparathyroidism [7]. However, to date, there are no clear data elucidating how these skeletal features could potentially affect bone strength and influence bone fragility, and no clear data on fragility fracture prevalence or fracture risk in HypoPT are available yet. In this light, any study that evaluates the state of bone health and the occurrence of fragility fractures in cohorts of HypoPT patients is important to increase and deepen clinical evidences in this area, to confirm and strengthen the results obtained in previous studies, and to help including also this specific clinical aspect in the life-long management of HypoPT patients.

Data from case–control studies are conflicting, finding no difference in overall fracture rate with respect to the general population [4, 8], or showing a higher prevalence of vertebral fractures in idiopathic HypoPT than in healthy subjects [9] and in HypoPT postmenopausal women than in matched controls [10, 11], and of rib fractures in patients with post-surgical permanent HypoPT than in thyroidectomized normocalcemic control patients [8]. A very recent study on the Canadian National Hypoparathyroidism Registry found a fragility fracture overall prevalence of 11.9% in the 101 (18 men, 35 premenopausal, and 48 postmenopausal women) analyzed HypoPT cases, the great majority (over 83%; 10/12) of them being postmenopausal women [12]. No fragility fractures or low BMD were reported in premenopausal women.

In this cross-sectional study, we retrospectively investigated bone status in a cohort of HypoPT patients from the Italian HypoparaNET database [2, 12], for which a DXA analysis was available, based on their sex, age, HypoPT etiology, urinary excretion of calcium and phosphate, and serum level of 25(OH)-vitamin D.

## Patients and methods

### Patients

The HypoparaNET database was the first instituted Italian cross-sectional multicenter database of patients with HypoPT [2, 13]. From March 2014 to September 2015, it collected data from 20 Clinical Centers in Italy (16 Endocrinology and 4 Endocrine Surgery Centers), with the first clinical evaluation at each recruiting clinical center varying between 1980 and September 2015. The study was approved by the Local Ethical Committee of the Coordinator Center in Florence (Comitato Etico Area Vasta Centro, Florence, Italy) [study reference number: 10641_oss]. All the enrolled patients signed a specific informed consent form before their baseline data were retrospectively and anonymously retrieved by their medical records and collected in the HypoparaNET database. Collected baseline data, analyzed in the present study, were referred to the first visit performed at the recruiting clinical center, differing from time of HypoPT diagnosis and from onset of the disease.

The HypoparaNet database includes 509 patients with HypoPT (399 women and 110 men). Patients had a mean age of 41.9 ± 19.5 years (median 43.0; range 0–89 years) at the time of HypoPT onset, and a mean age at clinical data collection of 48.3 ± 19.7 years (median 52.0; range 0–94 years).

HypoPT etiology included 363 (71.3%) post-surgical, 78 (15.3%) idiopathic and 64 (12.6%) genetically-determined cases; the remaining 4 HypoPT cases were due to neck irradiation or parathyroid tissue infiltration (i.e. tuberculosis). Of the post-surgical HypoPT, 304 were due to total thyroidectomy, 13 were caused by partial thyroidectomy, and 30 were subsequent to parathyroidectomy (6 single gland excisions, 24 removals of more than 2 glands, and 16 cases for which the primary neck surgery was not reported in the database).

### Bone status

For the analysis of bone status in the HypoparaNET population, we retrieved the available DXA scan performed at least at one of the following 4 skeletal sites: lumbar spine (L1–L4), femur neck, total femur, and non-dominant distal radius. Data were specifically analyzed in the following population subsets:

- Total HypoPT cases > 19 years (adult HypoPT cases).
- HypoPT cases ≤ 19 years (children and adolescents with HypoPT).
- HypoPT women vs HypoPT men (both > 19 years).
- HypoPT cases 20–50 years vs HypoPT cases > 50 years.
- HypoPT women 20–50 years or pre-menopausal vs HypoPT women > 50 years or postmenopausal.
- HypoPT men 20–50 years vs HypoPT men > 50 years.
- Different etiologies of HypoPT (i.e. post-surgical, genetically-determined, idiopathic).
- Adult HypoPT cases with normal 24 h urinary excretion of calcium (≤ 300 mg/24 h) vs adult HypoPT cases with hypercalciuria (> 300 mg/24 h)..
- Adult HypoPT cases with normal 24 h urinary excretion of phosphate (≥ 400 mg/24 h) vs adult HypoPT cases with hypophosphaturia (< 400 mg/24 h).
- Adult HypoPT cases with deficiency/insufficiency of 25(OH)-vitamin D (< 30 ng/ml) vs adult HypoPT cases with sufficient levels of 25(OH)-vitamin D (≥ 30 ng/ml).

According to DXA scores and the diagnostic criteria of the World Health Organization (WHO), adult HypoPT cases > 19 years were categorized based on their bone status as follows [14]:

- Osteoporosis if at least one of the measured skeletal sites had a T-score ≤ − 2.5.
- Osteopenia if at least one of the measured skeletal sites had a T-score < − 1.0, and all the measured skeletal sites were > − 2.5.
- Normal BMD if all the measured skeletal sites had T-scores ≥ − 1.0 and ≤  + 2.0.
- Increased BMD if at least one of the measured bone sites had a T-score >  + 2.0.

For this classification, T-score values (standard deviation difference of patients’ BMD with respect to the mean BMD value of the healthy 30-year-old reference population) were considered for men over 50 years of age and postmenopausal women, while Z-score values (standard deviation difference of patients’ BMD with respect to the mean BMD value of a healthy population of the same age and gender) were used for premenopausal women and men younger than 50 years.

In children and adolescents up to 19 years, bone status was classified as it follows [15, 16]:

- Reduced bone mass for their age if at least one of the measured skeletal sites had a Z-score ≤ − 2.0
- Normal bone mass for their age if all the measured skeletal sites had Z-scores > − 2.0
- Increased BMD if at least one of the measured bone sites had a T-score >  + 2.0

In 39 HypoPT cases, a total-body skeletal X-ray exam was available, primarily performed to assess the presence of ectopic calcifications. These radiological data were used to assess absence or presence of vertebral fractures, using a morphometric qualitative and quantitative evaluation of vertebral shape according to Genant method.

### Analyses

Descriptive and statistical analyses of bone data were performed on the parameters of interest in the population subsets described in subSect. “Bone status”.

Continuous variables were reported in the form of mean ± standard deviation (SD) and median with minimum and maximum values (range), while categorial variables were expressed as percentages.

Statistical comparisons of continuous variables between population subsets were performed by using the Student’s t-test. Differences in prevalence of categorial variables between population subsets were analyzed using the χ^2^ test. For both statistical tests a p value less than 0.05 was assumed as indicator of statistical significance (over 95% likelihood that a difference between two variable is true).

All statistical analyses were performed using a website resource for social science statistic (freely available at https://www.socscistatistics.com/).

## Results

DXA parameters for at least one of the 4 skeletal sites of interest were available in 173 of 509 HypoPT cases (34.0%), including 142 women and 31 men. Of them 11 were children or adolescents up to the age of 19 years (7 females and 4 males) and 162 were adults aged over 20 years (135 women and 27 men). The adult cohort included 38 women ≤ 50 years or premenopausal, 97 women > 50 years or postmenopausal, 13 men ≤ 50 years, and 14 men > 50 years.

At the time of DXA analysis, 147 patients were in treatment with both calcium and vitamin D replacement therapy, 17 were taking only vitamin D supplementation, and 5 only calcium. Four patients were not reported to take either calcium or vitamin D. In addition, 4 patients were also treated by PTH 1–34 (teriparatide), two of them (one man aged 28 years and one premenopausal woman aged 48 years) receiving 20 mcg/day, and two of them (one man aged 53 years and a woman aged 37 years) receiving 40 mcg/day. Three of them having normal BMD values both at femur and lumbar spine and the 48-year-old woman having normal BMD at femur and osteopenia at lumbar spine. Finally, one 19-year-old female patient with a genetic form of HypoPT was under therapy with PTH 1–84 (4.0 mcg/day) presenting normal BMD (according to the Z-scores) at all the three measured bone sites.

Detailed prevalence of osteoporosis, osteopenia, normal BMD, and increased BMD in adult HypoPT cases, and of reduced, normal or increased BMD for age and sex in children and adolescent HypoPT cases are reported in Table 1, overall and in specific population subsets.

**Table 1:** Prevalence of different bone status in our HypoPT patients

HypoPT cases	DXA evaluation				
	No. cases in which DXA analysis was available* (%)	Osteoporosis (no. cases) (%)	Osteopenia (no. cases) (%)	Normal BMD (no. cases) (%)	Increased BMD (no. cases) (%)
Total HypoPT cases > 19 years (n = 453)	162 (35.8)	28 (17.3) Mean age 62.3 ± 12.2 years (median 65.0; range 28–78)	50 (30.9) Mean age 56.0 ± 14.0 years (median 60.5; range 20–81)	60 (37.0.) Mean age 49.6 ± 14.4 years (median 52.0; range 20–75)	24 (14.8) Mena age 55.0 ± 16.7 years (median 55.0; range 24–80)
Total HypoPT cases ≤ 19 years (n = 56)	11 (19.6)	Not applicable	2 (18.2) Aged 6 and 10 years	7 (63.6) Mean age 12.9 ± 6.8 years (median 14.0; range 4–19)	2 (18.2.) Aged 17 and 19 years
HypoPT cases 20–50 years (n = 183)	51 (27.9)	4 (7.8) Mean age 38.3 ± 7.3 years (median 40.5; range 28–44)	14 (27.5) Mean age 37.8 ± 10.8 years (median 41.0; range 20–50)	27 (52.9) Mean age 36.0 ± 8.2 years (median 36.0; range 20–49)	6 (11.8) Mean age 37.5 ± 7.4 years (median 38.0; range 24–46)
HypoPT cases > 50 years (n = 270)	111 (41.1)	24 (21.6) Mean age 66.3 ± 7.2 years (median 68.0; range 54–78)	36 (32.4) Mean age 63.1 ± 6.9 years (median 63.5; range 51–81)	33 (29.7) Mean age 60.7 ± 6.6 years (median 61.0; range 51–75)	18 (16.2) Mean age 60.8 ± 8.0 years (median 59.5; range 51–75)
Total HypoPT women > 19 years (n = 362)	135 (37.3)	25 (18.5) Mean age 64.3 ± 10.1 years (median 68.0; range 38–78)	46 (34.1) Mean age 56.1 ± 13.3 years (median 59.5; range 23–81)	47 (34.8) Mean age 52.0 ± 12.6 years (median 53.0; range 27–75)	17 (12.6) Mean age 55.5 ± 18.9 years (median 55.0; range 24–80)
HypoPT women 20–50 years/premenopausal (n = 140)	38 (27.1)	2 (5.3) Aged 38 and 43 years	13 (34.2) Mean age 39.2 ± 9.9 years (median 43.0; range 23–50)	19(50.0) Mean age 39.2 ± 7.0 years (median 41.0; range 27–49)	4 (10.5) Mean age 36.0 ± 9.1 years (median 37.0; range 24–46)
HypoPT women > 50 years/postmenopausal (n = 222)	97 (43.7)	23 (23.7) Mean age 66.4 ± 7.3 years (median 68.0; range 54–78)	33 (34.0) Mean age 62.8 ± 7.1 years (median 62.0; range 51–81)	28 (28.9) Mean age 60.7 ± 6.7 years (median 61.0; range 51–75)	13 (13.4) Mean age 61.5 ± 8.8 years (median 60.0; range 51–80)
Total HypoPT men > 19 years (n = 91)	27 (29.7)	3 (11.1) Mean age 45.7 ± 18.6 years (median 44.0; range 28–65)	4 (14.8) Mean age 55.3 ± 23.6 years (median 65.5; range 20–70)	13 (48.2) Mean age 40.9 ± 17.5 years (median 34.0; range 20–68)	7 (25.9) Mean age 53.7 + 21.2 years (median 55.0; range 39–67)
HypoPT men 20–50 years (n = 43)	13 (30.2)	2 (15.4) Aged 28 and 44 years	1 (7.7) Aged 20 years	8 (61.5) Mean age 28.4 ± 5.2 years (median 27.5; range 20–37)	2 (15.4) Aged 39 and 42 years
HypoPT men > 50 years (n = 48)	14 (29.2)	1 (7.1) Aged 65 years	3 (21.4) Mean age 67.0 ± 2.6 years (median 66.0; range 65–70)	5 (35.7) Mean age 61.0 ± 7.0 years (median 64.0; range 53–68)	5 (35.7) Mean age 59.0 ± 5.7 years (median 56.0; range 54–67)
Post-surgical HypoPT cases (n = 363)	127 (35.0)	24 (18.9) Mean age 65.4 ± 8.6 years (median 68.0; range 43–78)	44 (34.6) Mean age 56.2 ± 13.2 years (median 59.5; range 23–81)	44 (34.6) Mean age 53.3 ± 12.6 years (median 54.0; range 20–75)	15 (11.8) Mean age 56.5 ± 13.1 years (median 55.0; range 37–80)
Idiopathic HypoPT cases (n = 78)	29 (37.2)	1 (3.4) Aged 65 years	4 (13.8) Mean age 60.0 ± 14.2 years (median 65.5; range 39–70)	14 (48.3) Mean age 34.8 ± 17.0 years (median 29.5; range 11–68)	10 (34.5) Mean age 49.2 ± 16.1 years (median 54.5; range 19–67)
Genetically-determined HypoPT cases (n = 64)	14 (21.9)	0	8 (57.1) Mean age 18.1 ± 21.1 years (median 12.0; range 4–68)	5 (35.7) Mean age 38.0 ± 12.2 years (median 33.0; range 26–53)	1 (7.1) Aged 17 years
HypoPT cases from other causes (n = 4)	3 (75)	3 (100) Mean age 36.7 ± 8.1 years (median 38.0; range 28–44)	0	0	0
Total hypoPT cases with normal values of calciuria (≤ 300 mg/24 h) (n = 160)	87 (54.4)	11 (12.6) Mean age 64.7 ± 12.3 years (median 71.0; range 38–78)	28 (32.2) Mean age 52.5 ± 16.9 years (median 60.0; range 6–67)	32 (36.8) Mean age 48.8 ± 16.2 years (median 50.0; range 19–75)	16 (18.4) Mean age 55.1 ± 12.1 years (median 55.0; range 24–71)
Total hypoPT cases with hypercalciuria (> 300 mg/24 h) (n = 71)	33 (46.5)	7 (21.2) Mean age 60.1 ± 12.3 years (median 68.0; range 43–72)	8 (24.2) Mean age 63.9 ± 11.3 years (median 61.5; range 50–81)	11 (33.3) Mean age 49.3 ± 14.2 years (median 55.0; range 27–66)	7 (21.2) Mean age 42.7 ± 20.4 years (median 39.0; range 17–68)
HypoPT cases > 19 years with normal values of calciuria (≤ 300 mg/24 h) (n = 151)	83 (55.0)	11 (13.3) Mean age 64.7 ± 12.3 years (median 71.0; range 38–78)	26 (31.3) Mean age 55.9 ± 11.8 years (median 61.5; range 23–67)	30 (36.1) Mean age 50.8 ± 14.7 years (median 51.0; range 26–75)	16 (19.3) Mean age 55.1 ± 12.1 years (median 55.0; range 24–71)
HypoPT cases aged 20–50 years with normal values of calciuria (≤ 300 mg/24 h) (n = 65)	26 (40.0)	1 (3.8) Aged 38 years	8 (30.8) Mean age 41.0 ± 8.8 years (median 44.0; range 23–49)	14 (53.8) Mean age 37.7 ± 8.4 years (median 39.0; range 26–49)	3 (11.5) Mean age 35.7 ± 11.1 years (median 37.0; range 24–46)
HypoPT cases > 50 years years with normal values of calciuria (≤ 300 mg/24 h) (n = 86)	57 (66.3)	10 (17.5) Mean age 67.4 ± 9.0 years (median 71.0; range 56–78)	18 (31.6) Mean age 62.5 ± 4.6 years (median 64.5; range 52–67)	16 (28.1) Mean age 62.3 ± 7.6 years (median 64.5; range 51–75)	13 (22.8) Mean age 59.5 ± 6.8 years (median 59.0; range 51–71)
HypoPT cases > 19 years with hypercalciuria (> 300 mg/24 h) (n = 65)	31 (47.7)	7 (22.6) Mean age 60.1 ± 12.3 years (median 68.0; range 43–72)	8 (26.7) Mean age 63.9 ± 11.3 years (median 61.5; range 50–81)	11 (35.5) Mean age 49.3 ± 14.2 years (median 55.0; range 27–66)	5 (16.1) Mean age 52.6 ± 14.0 years (median 56.0; range 37–68)
HypoPT cases aged 20–50 years with hypercalciuria (> 300 mg/24 h) (n = 27)	9 (33.3)	2 (22.2) Aged 43 and 44 years	1 (11.1) Aged 50 years	4 (44.4) Mean age 32.8 ± 6.9 years (median 31.0; range 27–42)	2 (22.2) Aged 37 and 39 years
HypoPT cases > 50 years with hypercalciuria (> 300 mg/24 h) (n = 38)	22 (57.9)	5 (22.7) Mean age 63.9 ± 5.7 years (median 68.0; range 57–72)	7 (31.8) Mean age 65.9 ± 10.6 years (median 62.0; range 51–81)	7 (31.8) Mean age 58.7 ± 5.1 years (median 57.0; range 52–66)	3 (13.6) Mean age 62.3 ± 6.0 years (median 63.0; range 56–68)
Total hypoPT cases with normal values of phosphaturia (≥ 400 mg/24 h) (n = 101)	60 (59.4)	4 (6.7) Mean age 56.5 ± 1.6 years (median 56.0; range 43–71)	14 (23.3) Mean age 55.9 ± 12.9 years (median 57.0; range 34–81)	29 (48.3) Mean age 52.2 ± 14.1 years (median 52.0; range 26–75)	13 (21.7) Mean age 44.1 ± 16.2 years (median 53.0; range 17–63)
Total hypoPT cases with hypophosphaturia (< 400 mg/24 h) (n = 61)	34 (55.7)	6 (17.6) Mean age 62.5 ± 15.3 years (median 65.5; range 38–74)	12 (35.3) Mean age 57.7 ± 14.5 years (median 62.0; range 23–78)	9 (26.5) Mean age 41.7 ± 18.6 years (median 41.0; range 19–68)	7 (20.6) Mean age 59.6 ± 9.8 years (median 60.0; range 46–71)
HypoPT cases > 19 years with normal values of phosphaturia (≥ 400 mg/24 h) (n = 93)	58 (62.4)	4 (6.9) Mean age 56.5 ± 15.1 years (median 56.0; range 43–71)	14 (24.1) Mean age 55.9 ± 12.9 years (median 57.0; range 34–81)	29 (50.0) Mean age 52.2 ± 14.1 years (median 52.0; range 26–75)	11 (19.0) Mean age 48.8 ± 12.5 years (median 54.0; range 24–63)
HypoPT cases aged 20–50 years with normal values of phosphaturia (≥ 400 mg/24 h) (n = 43)	23 (53.5)	2 (8.7) Aged 43 and 44 years	5 (21.7) Mean age 42.6 ± 6.3 years (median 43.0; range 34–50)	12 (52.2) Mean age 38.2 ± 8.2 years (median 39.5; range 26–49)	4 (17.4) Mean age 34.3 ± 6.9 years (median 37.0; range 24–39)
HypoPT cases > 50 years with normal values of phosphaturia (≥ 400 mg/24 h) (n = 50)	35 (70.0)	2 (5.7) Aged 68 and 71 years	9 (25.7) Mean age 63.2 ± 8.9 years (median 62.0; range 51–81)	17 (48.6) Mean age 62.1 ± 7.0 years (median 64.0; range 51–75)	7 (20.0) Mean age 57.1 ± 3.6 years (median 56.0; range 53–63)
HypoPT cases > 19 years with hypophosphaturia (< 400 mg/24 h) (n = 57)	32 (56.1)	6 (18.7) Mean age 62.5 ± 15.3 years (median 65.5; range 38–78)	12 (37.5) Mean age 57.7 ± 14.5 years (median 62.0; range 23–78)	7 (21.9) Mean age 48.1 ± 15.6 years (median 45.0; range 28–68)	7 (21.9) Mean age 59.6 ± 9.8 years (median 59.8; range 46–71)
HypoPT cases aged 20–50 years with hypophosphaturia (< 400 mg/24 h) (n = 25)	10 (40.0)	1 (10.0) Aged 38 years	4 (40.0) Mean age 41.3 ± 12.3 years (median 46.5; range 23–49)	4 (40.0) Mean age 36.8 ± 7.7 years (median 37.0; range 28–45)	1 (10.0) Aged 46 years
HypoPT cases > 50 years with hypophosphaturia (< 400 mg/24 h) (n = 32)	22 (68.8)	5 (22.7) Mean age 67.4 ± 10.7 years (median 73.0; range 54–78)	8 (36.4) Mean age 65.9 ± 5.8 years (median 65.5; range 59–78)	3 (13.6) Mean age 63.3 ± 5.7 years (median 65.0; range 57–68)	6 (27.3) Mean age 61.8 ± 8.5 years (median 61.8; range 51–71)
Total hypoPT cases with 25(OH)-vitamin D deficiency/insufficiency (< 30 ng/ml) (n = 124)	52 (41.9)	12 (23.1) Mean age 60.0 ± 13.2 years (median 60.0; range 68–72)	14 (26.9) Mean age 57.7 ± 16.1 years (median 62.0; range 10–78)	16 (30.8) Mean age 40.0 ± 21.2 years (median 33.5; range 4–75)	10 (19.2) Mean age 59.5 ± 9.9 years (median 57.5; range 46–80)
Total hypoPT cases with normal levels of 25(OH)-vitamin D (≥ 30 ng/ml) (n = 98)	40 (40.8)	3 (7.5) Mean age 69.7 ± 2.1 years (median 69.0; range 68–72)	14 (35.0) Mean age 56.0 ± 12.8 years (median 57.5; range 23–77)	18 (45.0) Mean age 47.0 ± 14.7 years (median 48.0; range 19–68)	5 (12.5) Mean age 48.8 ± 15.8 years (median 39.0; range 37–71)
HypoPT cases > 19 years with 25(OH)-vitamin D deficiency/insufficiency (< 30 ng/ml) (n = 113)	48 (42.5)	12 (25.0) Mean age 60.0 ± 13.2 years (median 60.0; range 38–78)	13 (27.1) Mean age 61.4 ± 8.7 years (median 63.0; range 48–78)	13 (27.1) Mean age 46.6 ± 17.4 years (median 53.0; range 26–75)	10 (20.8) Mean age 59.5 ± 9.9 years (median 57.5; range 46–80)
HypoPT cases aged 20–50 years with 25(OH)-vitamin D deficiency/insufficiency (< 30 ng/ml) (n = 46)	12 (26.1)	3 (25.0) Mean age 41.7 ± 3.2 years (median 43.0; range 38–44)	2 (16.7) Aged 48 and 49 years	6 (50.0) Mean age 29.8 ± 3.3 years (median 29.5; range 26–34)	1 (8.3) Aged 46 years
HypoPT cases > 50 years with 25(OH)-vitamin D deficiency/insufficiency (< 30 ng/ml) (n = 67)	36 (53.7)	9 (25.0) Mean age 66.1 ± 8.4 years (median 68.0; range 54–78)	11 (30.6) Mean age 63.7 ± 7.1 years (median 65.0; range 51–78)	7 (19.4) Mean age 61.0 ± 8.4 years (median 57.0; range 53–75)	9 (25.0) Mean age 61.0 ± 9.2 years (median 59.0; range 53–80)
HypoPT cases > 19 years with normal levels of 25(OH)-vitamin D (≥ 30 ng/ml) (n = 92)	38 (41.3)	3 (7.9) Mean age 69.7 ± 2.1 years (median 69.0; range 68–72)	14 (38.9) Mean age 56.0 ± 12.8 years (median 57.5; range 23–77)	16 (42.1) Mean age 50.5 ± 11.3 years (median 48.5; range 33–68)	5 (13.1) Mean age 48.8 ± 15.8 years (median 39.0; range 37–71)
HypoPT cases aged 20–50 years with normal levels of 25(OH)-vitamin D (≥ 30 ng/ml) (n = 44)	16 (36.4)	0	4 (25.0) Mean age 41.5 ± 12.5 years (median 46.5; range 23–50)	9 (56.3) Mean age 42.4 ± 6.5 years (median 45.0; range 33–49)	3 (18.7) Mean age 37.7 ± 1.2 years (median 37.0; range 37–39)
HypoPT cases > 50 years with normal levels of 25(OH)-vitamin D (≥ 30 ng/ml) (n = 48)	22 (41.7)	3 (13.6) Mean age 69.7 ± 2.1 years (median 69.0; range 68–72)	10 (45.5) Mean age 61.8 ± 7.2 years (median 62.0; range 52–77)	7 (31.8) Mean age 60.9 ± 6.2 years (median 64.0; range 51–68)	2 (9.1) Aged 60 and 71 years

* = Availability of at least one T-score value (or Z-score value for individuals aged 20–50 years, and for children and adolescents) in one of the four bone sites (lumbar spine, femur neck, total femur, and distal radius)

Numbers in parentheses, in columns 2–6, indicate a percentage value

% of osteoporosis, osteopenia, normal BMD, and increased BMD were calculated respect to the number of HypoPT cases with available DXA evaluation. For children and adolescents up to 19 years reduced bone mass (osteopenia), normal bone mass and increased bone mass, according to their age reference values, have been assessed

### Bone status according to ageing and sex

Bone mass loss was positively associated with both ageing and female gender.

Indeed, osteoporosis affected 21.6% of HypoPT patients over 50 years of age, but only 6.4% of those aged 20–50 years (χ^2^ = 4.64; p value = 0.0312; odds ratio (OR) = 0.31). Comparison between women and men showed that both osteoporosis (18.5% vs 11.1%; χ^2^ = 0.86; p value = 0.353; OR = 0.55) and osteopenia (34.1% vs 14.8%; χ^2^ = 3.91; p value = 0.048; OR = 0.34) were more frequent in women, reaching a slightly significant difference only for osteopenia.

### Bone status according to menopause (estrogen deficiency)

Interestingly, when we specifically assessed the prevalence of osteopenia/osteoporosis in women in relation to menopause (or age 20–50 years or above 50 years), we found that the prevalence of osteopenia was high and comparable in the F ≤ 50 and F > 50 subgroups, averaging 34.2% and 34.0%, respectively (χ^2^ = 0.00; p value = 0.983; OR = 1.01). The prevalence of osteoporosis was clearly and significantly higher in the F > 50 group as compared to the F ≤ 50 group, averaging 23.7% and 5.3% respectively (χ^2^ = 6.16; p value = 0.013; OR = 0.18).

### Bone status according to HypoPT etiology

Based on the HypoPT etiology, the prevalence of osteoporosis and osteopenia, or reduced bone mass for age and sex, was significantly higher in patients with post-surgical HypoPT than in those with idiopathic HypoPT (respectively 18.9% vs 3.4%; χ^2^ = 4.19; p value = 0.041; OR = 6.52 and 34.6% vs 13.8%; χ^2^ = 4.82; p value = 0.028; OR = 3.31). Conversely, patients with idiopathic HypoPT had a significantly higher prevalence of increased BMD than those with post-surgical HypoPT (34.5% vs 11.8%; χ^2^ = 9.02; p value = 0.003; OR = 0.25). Age at DXA evaluation was significantly higher (t-test = 4.396; p value = 0.00002) in patients with post-surgical HypoPT (mean 57.0 ± 12.9 years; median 59.0; range 20–81 years) than in those with idiopathic HypoPT (mean 44.3 ± 18.5 years; median 42.0; range 11–70 years). Despite the very young age at DXA evaluation for the 14 cases of genetic HypoPT (mean 25.1 ± 19.6 years; median 19.5; range 4–68 years), these patients showed an overall high prevalence of osteopenia or reduced bone mass according to their age and sex (57.1%).

### Bone status according to urinary calcium and phosphate excretion

In adult cases, no significant differences in the prevalence of osteoporosis, osteopenia, normal BMD, or increased BMD were found between patients with normal calciuria vs patients with hypercalciuria (p values = 0.224, 0.567, 0.948 and 0.700, respectively). No significant differences in the prevalence of osteoporosis, osteopenia, or increased BMD were also found between patients with normal phosphaturia vs patients with hypophosphaturia (p values = 0.087, 0.181, 0.741, respectively), while frequency of normal BMD resulted to be significantly higher in patients with normal phosphaturia (50.0% vs 21.9%; χ^2^ = 6.80; p value = 0.009; OR = 3.57).

### Bone status according to vitamin D levels

The status of deficiency/insufficiency of 25(OH)-vitamin D was significantly associated with osteoporosis with respect to adult patients with sufficient levels of 25(OH)-vitamin D (25.0% vs 7.9%; χ^2^ = 4.31; p value = 0.038; OR = 3.89). No significant differences were instead found for osteopenia, normal BMD, or increased BMD (p values = 0.333, 0.143, 0.352, respectively).

### Analysis of DXA scores

**Fig. 1:**
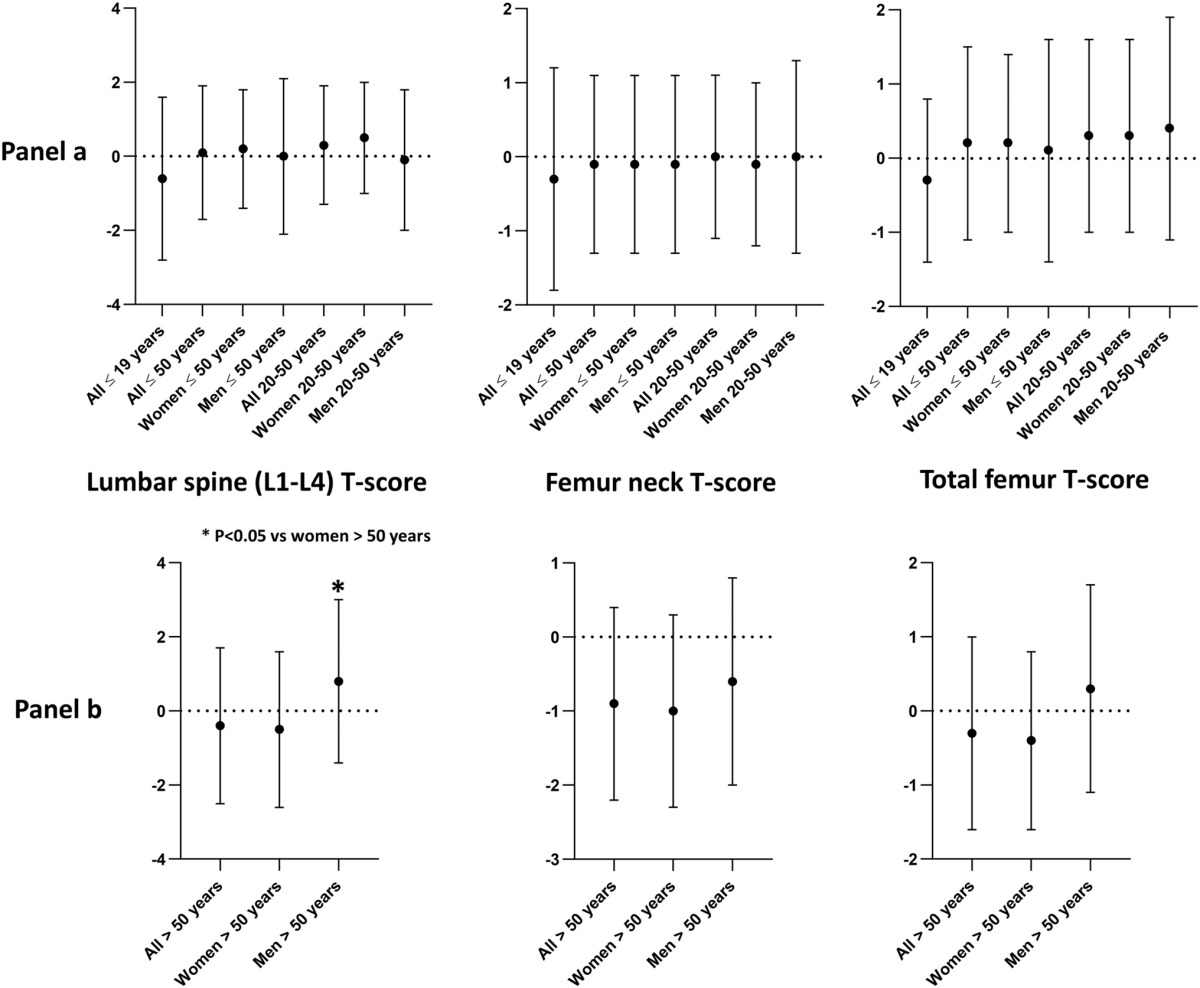
Comparison of mean values of DXA scores, measured at three different skeletal sites, in female and male HypoPT patients, according to the age. Panel a reports values of Z-score for HypoPT patients ≤ 50 years, while Panel b shows T-score for HypoPT patients > 50 years. Graphs have been generated by GraphPad Prism 8:4.2

Mean values of Z-score ± SD (for cases ≤ 50) and of T-score ± SD (for cases > 50), at three evaluated skeletal sites (lumbar spine, femur neck and total femur), are shown in Fig. 1.

Data on non-dominant distal radius were not reported since this skeletal site was used to assess bone status only in two of the 173 cases.

At lumbar spine, women over 50 had significantly lower mean T-scores than men over 50 (t-test = − 2.063; p value = 0.042). No significant differences were found at femur neck and total femur between different groups of HypoPT patients.

**Fig. 2:**
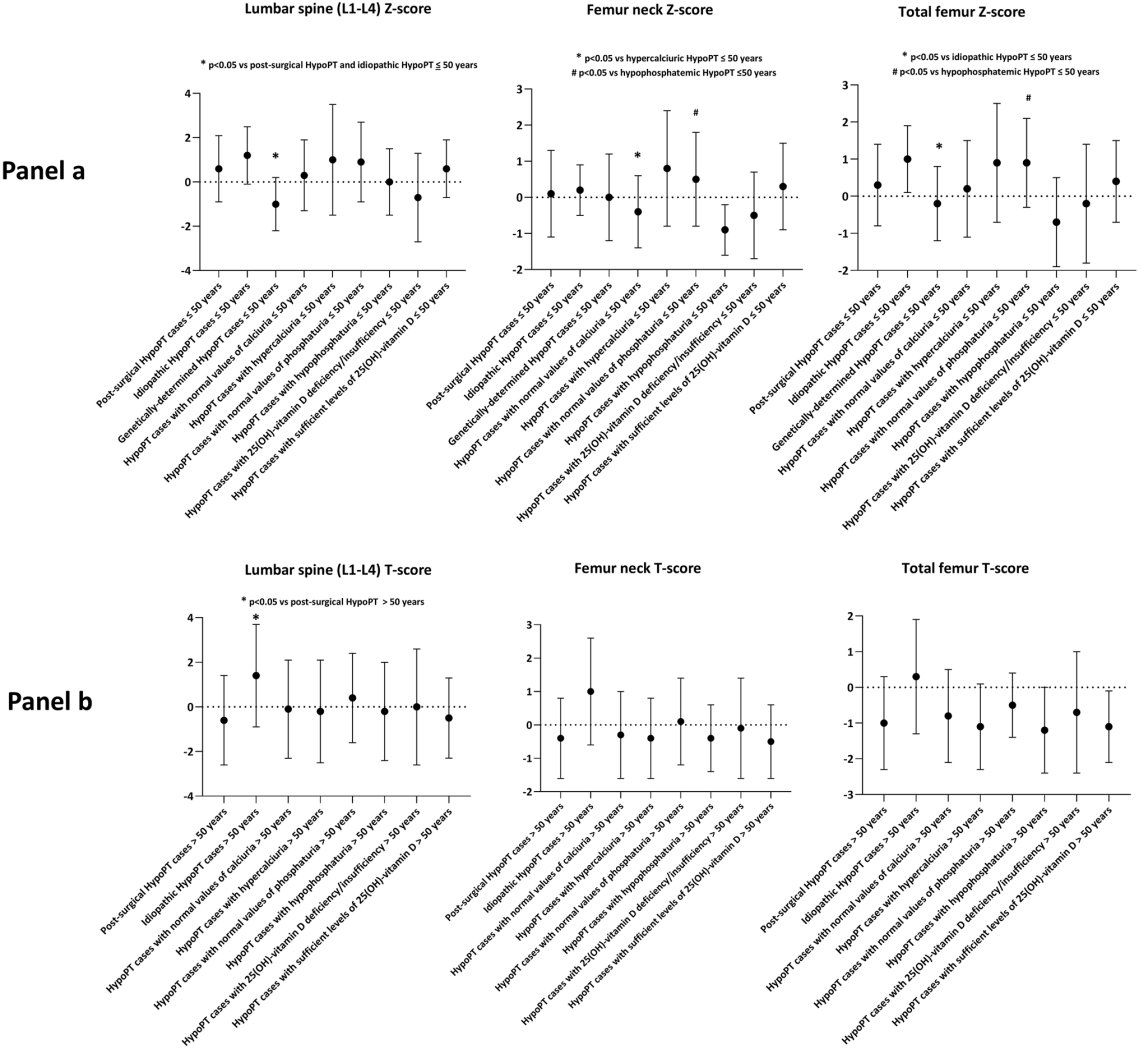
Comparison of mean values of DXA scores, measured at three different skeletal sites, in HypoPT population subsets ≤ 50 years or > 50 years, based on HypoPT etiology, urinary excretion of calcium and phosphate, and serum level of 25(OH)-vitamin D. Panel **a** reports values of Z-score for HypoPT population subsets ≤ 50 years, while Panel **b** shows T-score for HypoPT population subsets > 50 years. Graphs have been generated by GraphPad Prism 8:4.2

Comparisons of Z-score ± SD (for HypoPT population subsets ≤ 50 years) and of T-score ± SD (for HypoPT population subsets > 50 years), based on HypoPT etiology, urinary excretion of calcium and phosphate, and serum level of 25(OH)-vitamin D are shown in Fig. 2. Statistically significant differences (p value < 0.05) are indicated in the graphs.

Genetic HypoPT was associated with significantly lower mean value of Z-score at lumbar spine, but not at femur sites, than both post-surgical and idiopathic HypoPT, in individuals less than 50 years. These data cannot be confirmed in genetic HypoPT over 50 years since T-scores were available in only one genetic case. In individuals ≤ 50 years-old, both hypercalciuria and hypophosphaturia were positively associated with significantly lower mean value of Z-scores at femur neck and total femur, but not at lumbar spine. These associations were not confirmed in HypoPT over 50 years.

**Fig. 3:**
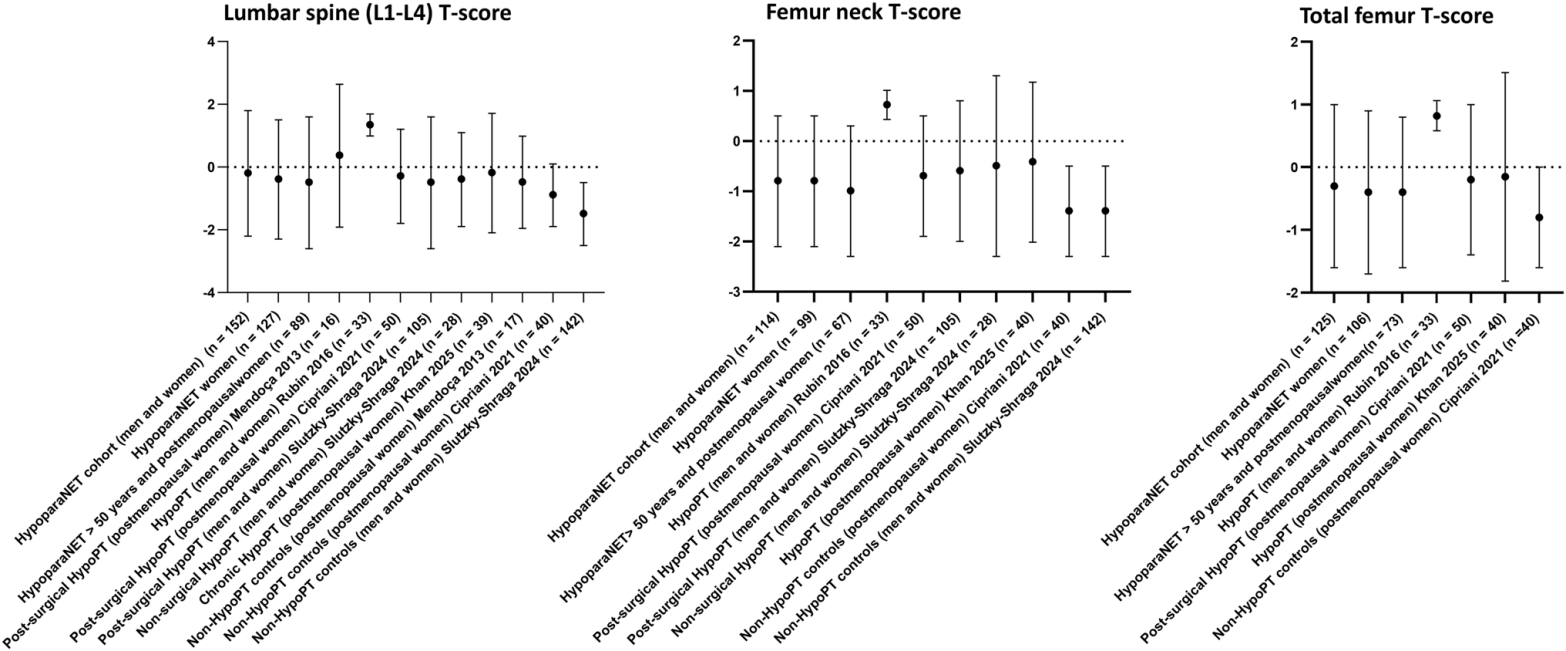
Comparison of mean values of T-scores, measured at three different skeletal sites in our HypoPT cohort with respect to HypoPT cases and non-HypoPT controls from previously published studies. Graphs have been generated by GraphPad Prism 8:4.2

We compared mean T-score values between our HypoPT cohort and data from previously published studies by Mendoça et al. [11], Cipriani et al. [10], Slutzky‑Shraga et al. [8], Khan et al. [12] and Rubin et al. [17] (Fig. 3), which included, respectively:

- 16 postmenopausal women with post-surgical HypoPT (mean age 62.3 ± 8.9 years), and 17 postmenopausal age-matched non-HypoPT controls (mean age 58.8 ± 6.1 years),
- 50 postmenopausal women with post-surgical HypoPT (mean age 65.4 ± 9.0 years), and 40 age-matched healthy postmenopausal women (mean age 64 ± 8.5 years),
- 105 post-surgical (87 women and 18 men, mean age 47 ± 13 years) and 28 non-post-surgical (10 women and 18 men, mean age 21 ± 10 years) HypoPT patients, and 142 patients with a normal calcium and PTH levels after thyroidectomy (117 women and 25 men, mean age 48 ± 15 years),
- 40 postmenopausal HypoPT women (mean age of this subpopulation was not indicated),
- 33 HypoPT cases (16 premenopausal women, 10 postmenopausal women, and 7 men; overall aged 47.0 ± 2.3 years), of whom 20 post-surgical patients, 12 autoimmune HypoPT, and one DiGeorge syndrome.

### Morphometric vertebral fractures

An X-ray exam of the vertebrae was available in only 39 HypoPT patients (7.7%), including 29 women and 10 men. Mean age at evaluation was 52.5 ± 21.8 years (median 61.0; range 1–78 years). Vertebral fractures were identified in 11 of the 39 screened patients (28.2%; 8 women and 3 men). Mean age at X-ray evaluation was 47.5 ± 23.7 years (median 55.0; range 1–78 years) for HypoPT patients without vertebral fracture and 65.4 ± 5.8 years (median 66.0; range 56–75 years) for fractured HypoPT patients, with a significant difference between the two groups of patients (t-test = − 2.452; p value = 0.019).

Of the 11 fractured patients:

- one man had traumatic crushing of a single dorsal vertebra (in presence of normal values of BMD at lumbar spine),
- five had atraumatic mild vertebral crushing, two with multiple and one with single thoracic vertebrae affected (associated with normal (T-score 0.8), increased (T-score 2.8) or osteoporotic (T-score − 2.6) BMD values at lumbar spine), one with multiple fracture at dorsal vertebrae (lumbar spine T-score − 2.4), and one with single fracture of L1 (lumbar spine T-score − 0.42),
- five had vertebral crushing > 20%, all being multiple fractures of dorsal vertebrae (three with lumbar spine T-score − 2.8), and two with normal lumbar spine BMD (T-scores − 0.4 and − 0.1, respectively).

Of the 10 patients with vertebral fragility fracture, 7 (70%) were postmenopausal women (64.6 ± 4.5 years; median 66.0; range 56-68 years), and 3 (30%) elderly men (65.3 ± 9.5 years; median 65.0; range 56-75 years).

## Discussion

Although the fact that current HypoPT guidelines [1] do not recommend systematic evaluation of bone status in affected patients, the authors believe that bone health is an important clinical aspect to consider. The skeleton of patients with HypoPT is exposed to both the HypoPT-induced alteration of bone microarchitecture and the natural bone mass loss associated with ageing and estrogen deficiency due to menopause.

In this cross-sectional study, we retrospectively analyzed available DXA screenings in a cohort of Italian HypoPT patients from the HypoparaNET database, generating a picture of bone status in 162 adult patients and 11 children and adolescents with HypoPT, based on their sex, age, HypoPT etiology, urinary excretion of calcium and phosphate, and serum level of 25(OH)-vitamin D. The availability of DXA evaluation in only one third of all HypoPT cases of our database confirmed that bone health assessment is a rather neglected clinical aspect in the management of patients with HypoPT, especially men of any age and premenopausal women.

In our HypoPT cohort, 17.3% of patients > 19 years had osteoporosis, a percentage that is slightly lower but comparable to the global prevalence of 18.3% in the general population. This percentage was reported in a recent comprehensive systematic review and meta-analysis, which analyzed 86 international studies published up to August 26, 2020, including a total of 103,334,579 individuals aged 15–105 years, of whom 800,457 were women, and mostly diagnosing osteoporosis only through DXA assessment of BMD [18]. When individuals were separately analyzed by sex, the meta-analysis revealed a global osteoporosis prevalence of 23.1% in women and 11.7% in men, higher than the prevalence observed in our HypoPT adult women (18.5%), and comparable to that found in our HypoPT adult men (11.1%).

Salari et al. [19] conducted a systematic review and meta-analysis on the global prevalence of osteoporosis in elderly individuals. Their analysis included 40 international studies published up to March 2020, with a total of 79,127 individuals aged 50–85 years. They found an overall osteoporosis prevalence of 21.7% in older adults, which is nearly identical to the 21.6% prevalence observed in our HypoPT patients > 50 years. A subset meta-analysis of 15 studies out of 40 specifically evaluated the prevalence of osteoporosis in elder individuals by sex, showing an osteoporosis prevalence of 12.5% in men and of 35.3% in women, both being higher with respect to the 7.1% and the 23.7% observed, respectively, in our HypoPT men > 50 years and our HypoPT women > 50 years. Data from our HypoPT cohort confirmed that this endocrine condition is associated with an overall lower loss of bone mass, when compared to the general population of the same sex and age. Our study showed that even in patients with HypoPT ageing and female gender were positively associated with BMD reduction, representing two independent risk factors for osteoporosis.

Despite few available DXA screenings in genetic HypoPT cases, the analysis of DXA scores in HypoPT patients, based on the etiology, suggested that genetic forms of the disease were significantly associated with an early-onset bone mass loss at lumbar spine in patients less than 50 years (mean age at DXA evaluation 25.1 ± 19.6 years). Only one genetic HypoPT case > 50 years was available, not allowing to confirm this association trend also in elder patients. These data suggest that young patients with genetic forms of HypoPT should be given special attention regarding periodic monitoring of bone status, especially at vertebrae level.

In HypoPT patients under the age of 50, both hypercalciuric and hypophosphaturic states were positively associated with significantly lower BMD Z-scores at femoral neck, but not at lumbar spine, than patients with normal values of urinary calcium and phosphate excretion. However, those two significant associations were not confirmed in HypoPT patients > 50 years. Given the fact that no significant differences were reported in the overall prevalence of reduced BMD (osteoporosis or osteopenia) between normocalciuric and hypercalciuric HypoPT cases and between normophosphaturic and hypophosphaturic HypoPT cases, further studies are surely needed to better define if hypercalciuria and hypophosphaturia may exert a potential contribute in bone loss in HypoPT individuals aged less than 50 years.

Comparison of T-score mean values between our HypoPT cohort and data from five previously published studies showed comparable data, in all the analyzed skeletal sites, with respect to HypoPT cases by Mendoça et al. [11], Cipriani et al. [10], Slutzky‑Shraga et al. [8], and Khan et al. [12]. Conversely, the study by Rubin et al. [17] showed much higher mean values of T-score in their HypoPT patients, at all the three evaluated skeletal sites, compared with our HypoPT cohort, the HypoPT groups from the other four studies, and the three non-HypoPT control groups. This discordant result could be due to the lower mean age of the HypoPT population of the study by Rubin et al.

Despite the very low percentage of HypoPT patients (less than 8%) of the HypoparaNET having available data on the absence/presence of vertebral fracture, our data confirmed that ageing is a major risk factor for the occurrence of vertebral fragility fracture also in HypoPT patients.

Our study has some limitations, mainly the fact that DXA assessment was available only in one third of HypoPT patients of the HypoparaNET database, and that the DXA was likely performed in those patients who required this information (e.g. because of high risk of osteoporosis due to previous fracture, age, etc.), and, thus, available data on bone status might overestimate the prevalence of osteoporosis and bone mass loss. Moreover, data were cross-sectionally collected more than 10 years ago when the HypoparaNET database was established, and no update on these patients is available since then. Indeed, the HypoparaNET was a single snapshot of HypoPT patients, recorded at one point in time during the first visit at the clinical center; no follow-up data were included. Morphometrical evaluation of possible presence of vertebral fractures was available in only in 7.7% HypoPT cases from the HypoparaNET database, and, in all these patients, data on fractures were secondarily derived from a total-body X-ray scan, primarily performed to assess extra skeletal calcifications. Moreover, data on biochemical markers of bone metabolism were totally missing in the database, not allowing to monitor the degree of bone remodeling in our HypoPT patients. Finally, being a retrospective study, analyzed sample sizes vary according to the availability of parameters of interest in the database, therefore, subsets of patients for different parameters do not correspond to the same sub-population.

However, despite the limitations, with this study we got real-world evidence indicating that about half of HypoPT adult women, who represent the majority of the HypoPT population, had osteopenia or osteoporosis, and that prevalence of osteoporosis notably increases after 50 years and with menopause. Our study indicated also that young patients with genetic forms of HypoPT are prone to have a reduced BMD compared to healthy population of the same age and sex. Therefore, according to the authors, a constant monitoring of bone health, both through periodic measurement of biochemical bone turnover markers and DXA evaluation should be considered in the management of HypoPT patients, especially postmenopausal women and young individuals with genetic forms of HypoPT. When available at the referral medical center, a bone evaluation by pQCT or high-resolution pQCT (HR-pQCT) is indicated to assess bone quality and microstructure, an important component of bone strength not captured by DXA measurements, and possibly also including a vertebral fracture assessment through X-ray or DXA spine morphometry, in order to early detect this potential, and often silent, complication. In the context of HypoPT, new treatment modalities (i.e. PTH replacement therapy) for this patient population should also aim at restoring a balanced bone turnover, preventing bone tissue alteration, and granting a better bone health at any age.

Institution of prospective multicenter databases and national registries of HypoPT patients, which include tailored monitoring strategies and follow-up, is need. The institution of a novel Italian retro-prospective multicenter database of HypoPT patients has been recently approved by a national ethical committee and it is already active in collecting cases. This database is aimed to overcome limitations of the present study and to deepen, among others, the study of bone status and fracture risk in HypoPT, through the inclusion of a specific section dedicated to the collection of data about bone metabolism, bone status, and fracture history. The novel database will collect cases in both cross-sectional and longitudinal manners and it will include also the collection of data on patients with end-organ PTH-resistance diseases.

## Data Availability

The dataset analyzed in the current study (HypoparaNET database) is available from the corresponding author upon reasonable request.
